# Responses of root physiological characteristics and resistance gene expression to infection by *Meloidogyne incognita* at different temperatures in tobacco

**DOI:** 10.3389/fpls.2025.1592335

**Published:** 2025-07-21

**Authors:** Zhixiao Yang, Qilong Chen, Rengang Wang, Yingchao Lin, Dejun Kong, Zhihong Wang, Xinxi He, Zhujun Han, Yushuang Guo, Haiqian Xia, Yi Cao

**Affiliations:** ^1^ Guizhou Provincial Key Laboratory for Tobacco Quality Improvement and Efficiency Enhancement, Guizhou Academy of Tobacco Science, Guiyang, China; ^2^ Luohe City Company, Henan Provincial Tobacco Company, Luohe, China; ^3^ Technology Center, China Tobacco Hunan Industrial Company, Ltd., Changsha, China; ^4^ Jian City Company, Jiangxi Provincial Tobacco Company, Jian, China

**Keywords:** *Meloidogyne incognita*, temperature, tobacco, root, physiological characteristics, resistance gene

## Abstract

*Meloidogyne incognita* (*M*. *incognita*) is a highly destructive species of *Meloidogyne* spp., characterized by its ability to cause root-knot nematode (RKN) disease, which is difficult to control and severely inhibits plant growth. Temperature is one of the primary factors affecting *M*. *incognita* infection. However, the precise underlying mechanisms have not yet been clarified. The present study aims is to further explore the temperature-influenced resistance mechanisms to *M*. *incognita*. Antioxidant enzyme activities, osmotic regulation substance contents, tissue structure changes, and expression of the resistance gene (*Rk*) in the roots of two tobacco varieties were analyzed under three temperatures (15°C, 25°C, and 35°C) via artificial inoculation. A *M*. *incognita*-resistant variety (NC95) and a susceptible variety (CBH) was selected as experimental materials. The results showed that the activities of peroxidase (POD) and catalase (CAT), as well as the contents of soluble sugar, proline, and hydroxyproline-rich glycoprotein (HRGP), increased to varying degrees under *M*. *incognita* infection, while superoxide dismutase (SOD) activity decreased. Notably, the activities of POD and CAT, along with the contents of soluble sugar, proline, and HRGP, were all higher in NC95 than in CBH. Meanwhile, antioxidant enzyme activities and osmotic substance contents in both varieties varied most at 25°C and least at 35°C. No giant cells or oocysts were observed in the root tissues of NC95 at any temperature, whereas numerous giant cells and oocysts were present in CBH. The number of giant cells in CBH was highest at 25°C compared to 15°C and 35°C, and the degree of lignification in NC95 was also greater at 25°C. In addition, *M*. *incognita* infection induced the expression of *Rk* gene in NC95, with expression levels at 25°C and 15°C higher than at 35°C. The results indicated that SOD activity and osmotic regulatory substance contents decreased in the roots of the susceptible variety under *M*. *incognita* infection, accompanied by the appearance of numerous giant cells in the xylem, contributing to susceptibility. Conversely, the resistant tobacco variety exhibited stronger capabilities in reactive oxygen species (ROS) scavenging and osmotic regulation, no significant changes in root tissue structure, and upregulated expression of the *Rk* gene, all of which contributed to infection inhibition. Compared with the observations at 25°C, *M*. *incognita* infectivity on tobacco roots was effectively reduced by 35°C due to increased antioxidant enzyme activities, enhanced osmotic regulatory substance contents, and well-maintained root tissue structure. Additionally, *Rk* gene expression was not inactivated but only reduced at 35 °C, and it remained effective in inhibiting *M*. *incognita* infection.

## 1 Introduction

*Meloidogyne* spp. is one of the most destructive plant-parasites, causing root-knot nematode (RKN) disease and infecting more than 5,500 plant species, including food crops, cash crops, oil crops, vegetables, and fruits (Kaloshian and Teixeira, 2019; Yuan et al., 2023). It also exhibits notable characteristics such as large population variation, strong pathogenicity, a diverse array of hosts, wide distribution range, and concealed damage (Niu et al., 2022; Siddique et al., 2022). Once *Meloidogyne* spp. infection occurs, giant cells are induced from root tissue cells, leading to the formation of root knots, followed by root atrophy and deformity, and ultimately inhibiting the absorption of water and nutrients by plants (Holbein et al., 2016). In addition, the aboveground parts show slow growth and development, yellowing of leaves, yield reduction by 10%–20%, and, in severe cases, losses exceeding 75% or even complete crop failure, ultimately resulting in significant economic losses (Bird and Kaloshian, 2003; Tapia-Vázquez et al., 2022). With adjustments to the agricultural planting structure in China, the area devoted to high-value-added cash crops has expanded, and the multiple cropping index has also increased, leading to a year-by-year aggravation of *Meloidogyne* spp. occurrence and damage (Vestergård, 2019). To date, there is no particularly effective method for the prevention and control of *Meloidogyne* spp.

Chemical methods are widely used in agricultural production due to their fast-acting protective effects (Wang et al., 2024a). However, the use of highly toxic chemical agents not only seriously affects plant quality (Kanwar et al., 2021), but also pollutes the ecological environment (Abd-Elgawad and Askary, 2020; Hu et al., 2023). Therefore, pollution-free control of *Meloidogyne* spp. has become a current research hotspot (Vashisth et al., 2024).

Temperature is a key factor affecting *Meloidogyne* spp. infection (Qin, 2022), and the optimal temperature for its growth and development ranges from 15°C to 30°C (Liu, 2000). Devaney (2006) found that warming had a positive enhanced effect on *Meloidogyne* spp. in a suitable environment but became lethal when the temperature exceeded the optimal range. Matute (2013) indicated that *Meloidogyne* spp. exhibited pronounced thermophobia, and nearly all *Meloidogyne* spp. were killed at 49°C within 10 min–15 min (Jiang, 2006). An increase in temperature can enhance plant transpiration, reduce soil moisture content, and ultimately alter the growth microenvironment of *Meloidogyne* spp (Briones et al., 1999). At the same time, it also clearly promotes root growth and increases the substrate supply for heterotrophic respiration, thereby influencing the *Meloidogyne* spp. community (Kardol et al., 2011). Bakonyi and Nagy (2000) confirmed that increasing soil temperature reduced the richness and density of the *Meloidogyne* spp. community and significantly influenced its diversity and trophic structure. *Meloidogyne* spp. is a type of pest that spreads through soil as its medium. Therefore, controlling soil temperature can effectively alter the living environment, influence infection, reduce plants damage, and ultimately contribute to the prevention and control of *Meloidogyne* spp. Katan et al. (1976) proposed using the insulating effect of plastic film covering the soil surface to warm the soil and thereby kill *Meloidogyne* spp. India has made remarkable progress in controlling *Meloidogyne* spp., benefiting from a climate characterized by high temperature and arid conditions (Ros et al., 2008). In addition, treating soil with high-temperature water can reduce the incidence of *Meloidogyne* spp. infection, achieving a control rate of over 60% in China (He et al., 2009).

Tobacco (*Nicotiana tabacum* L.) is an important economic crop worldwide. Since the discovery in 1892, *Meloidogyne* spp. have been among the main pathogens affecting tobacco production worldwide (Tong et al., 2022). The dominant species, *Meloidogyne incognita* (*M*. *incognita*), has caused significant damage and substantial losses in tobacco due to its widespread occurrence, severe pathogenicity, and difficulty of control (Sang et al., 2024; Shakeel et al., 2020; Sikandar et al., 2020). Currently, several studies have examined the physiological and biochemical responses of tobacco to *M*. *incognita* infection (Li et al., 2018a, b; Lu et al., 2023). Each species within *Meloidogyne* spp. has an optimal growth temperature, but studies on the effects of temperature on the infectivity of *M*. *incognita* remain limited.

To date, no resistance genes to *Meloidogyne* spp. related to tobacco have been cloned, and the resistance mechanism remains unknown. Different resistance genes to *Meloidogyne* spp. infection exist in resistant varieties, and resistance in tobacco is complex and diverse (Xu et al., 2023). The *Rk* gene is the only known resistance gene to *Meloidogyne* spp. in tobacco. It was first discovered in *Nicotiana tomentosa* (Yi et al., 1998) and confers resistance to *Meloidogyne* spp., particularly to *M*. *incognita* (Adamo et al., 2021). However, to date, this gene has not been cloned, which limits its application potential. In addition, plants can regulate their internal metabolic activity under pathogen infection through a series of physiological and biochemical responses, including antioxidant enzyme activities and osmotic regulation substance content, to adapt and resist infection, minimize damage, and maintain normal physiological functions. Therefore, this study investigated changes in physiological characteristics—including antioxidant enzyme activities, osmotic regulatory substance contents, tissue structure, and expression of the resistance gene *Rk*—in roots of two tobacco varieties with differing resistance, infected by *M*. *incognita* at different temperatures The aim was to further clarify the effects of temperature on *M*. *incognita* infectivity and promote a theoretical basis for pollution-free control and the cultivation of resistant varieties.

## 2 Materials and methods

### 2.1 Experimental materials

Two tobacco varieties with contrasting responses to *M*. *incognita* were selected in the experiments—NC95 (resistant) (Xu et al., 2019) and CBH (susceptible) (Li et al., 2017). Seeds of both varieties were cultivated using the floating seedling method and then transplanted into plastic pots ((27 × 33 × 21 cm, height × caliber × bottom diameter) containing high-temperature-sterilized sand soil (sand-to-soil ratio of 1:3) when five leaves emerged (55 days post-germination), with an average plant height of approximately 12 cm. Each pot contained a single plant.

*M*. *incognita* were propagated from roots of greenhouse-grown susceptible tomato plants (S*olanum lycopersicum* L. ‘Rutgers’) that had been inoculated with the nematode 90 days earlier. After propagation, the infected tomato roots were harvested, and the second-stage juvenile (J2) of *M*. *incognita* were extracted using 0.5% NaOCl, following the method described by Tan and Wu (2003), and then hatched at 26 °C in a constant-temperature incubator. Freshly hatched J2 were preserved in deionized water for further inoculation. In addition, Peter’s counting slides were used to quantify the nematodes under a light microscope (XSM-20, China) (Fraher et al., 2024).

### 2.2 Experimental design

Seedling of two tobacco varieties at the 10-leaf stage were inoculated at the roots with approximately 1,000 J2 in 5 mL of deionized water, while control seedlings were mock-inoculated with the same volume of deionized water.

Temperature treatments were applied after inoculation with *M*. *incognita*. Based on studies on temperature during the tobacco growth period in the field (Liu, 2003) and the effects of different temperatures on *M*. *incognita* activity (Cao et al., 2012), the temperatures in the present experiment were set to 15°C, 25°C, and 35°C. The experiment was conducted in growth chambers with a photosynthetic photon flux density of 350 μmol m^−2^ s^−1^. Relative humidity (RH) was maintained at 70% under a 14-h photoperiod. A randomized complete block design was used with three replicates, and 80 plants per treatment were included for each tobacco variety.

### 2.3 Test sampling

According to the method described by Wang et al. (2006), samples were collected at 0 d, 14 d, and 28 d after inoculation with *M*. *incognita*. For each treatment, three tobacco plants of similar size from each variety were randomly selected. The sampled roots were combined, washed with distilled water, immediately frozen in liquid nitrogen, and stored at −80°C until further analysis.

Three plants from each tobacco variety were randomly selected for each treatment at 28 d after inoculation. The roots were cleaned with distilled water, and small knotted roots were selected and stored in formaldehyde–alcohol–acetic acid (FAA) fixative for paraffin sectioning. Paraffin sections were observed using a Leica 301-185.104–00 microscope (Germany), and images were captured with an Olympus DP70 camera (Japan).

Three NC95 plants from each treatment were sampled at 0 d, 2 d, 7 d, 14 d, 21 d, and 28 d after inoculation. The roots were washed with distilled water, immediately frozen in liquid nitrogen, and stored at −80°C to determine the relative expression levels of the resistance gene (*Rk*).

### 2.4 Indicators and methods for determination

Superoxide dismutase (SOD) activity was measured as described by Wang et al. (2024b). Peroxidase (POD) activity was assayed according to Yingsanga et al. (2008). Catalase (CAT) activity was determined using the method of Lu et al. (2017). Proline and soluble sugar were measured as described by Zhai et al. (2016) and Li (2000), respectively. Hydroxyproline-rich glycoprotein (HRGP) content was measured according to the method of Kivirikko et al. (1967).

Primer sequence information for the *Rk* gene is listed in **Table 1**, and the *Actin* gene was used as an internal control (Zhang et al., 2023). Total RNA was extracted using RNAiso Reagent (TaKaRa Inc., Japan). After confirming an OD260/OD280 ratio of approximately 2.0, cDNA was synthesized using a TaKaRa reverse transcription kit, and the resulting cDNA was used as a template for qRT-PCR amplification. The qRT-PCR reaction conditions were as follows: one cycle at 94°C for 5 min (initial denaturation), followed by 40 cycles of denaturation at 94°C for 15 s, annealing at 60°C for 15 s, and extension at 72°C for 30 s, with a final extension at 72°C for 10 min (Li et al., 2018b). Each amplification reaction was repeated four times. Data were analyzed using the 2^−ΔΔCT^ method to calculate relative gene expression levels (Yang et al., 2022).

**Table 1:** Primer sequence of the target gene *Rk* for qRT-PCR analysis.

Gene name	Accession number	Sequence of primers	Products length(bp)
*NtRk*	KP164989	5’-ATGCACAACGCCACAGTGAT-3’ 5’-CCTGCAATGACTCCAGCAATC-3’	190
*NtActin*	X63603	5’-CCACACAGGTGTGATGGTTG-3’ 5’-GTGGCTAACACCATCACCAG-3’	367

### 2.5 Statistical analysis

The relative increase of each physiological indicator was calculated according to the method of Xu et al. (2008), using the formula: relative increase = (measured value in treatment group/measured value in control group) × 100%. The relative increase reflects the ability of each physiological indicator in roots under *M*. *incognita* inoculation to maintain normal growth. In addition, a greater the deviation from a value from “1” indicates a stronger influence of *M*. *incognita*, whereas, a smaller deviation indicates a lesser degree of influence.

All the measurements were conducted with three independent biological replicates per determination, and mean values were presented with standard errors. Data were analyzed using SPSS 21.0 (Statistical Software Package) with one-way ANOVA, and differences between means were separated using the least significant difference (LSD) test at a 0.05 probability level, following the method of Hosseini et al. (2022).

## 3 Results

### 3.1 Effects of *M*. *incognita* infection on POD activity at different temperatures

**Figure 1:**
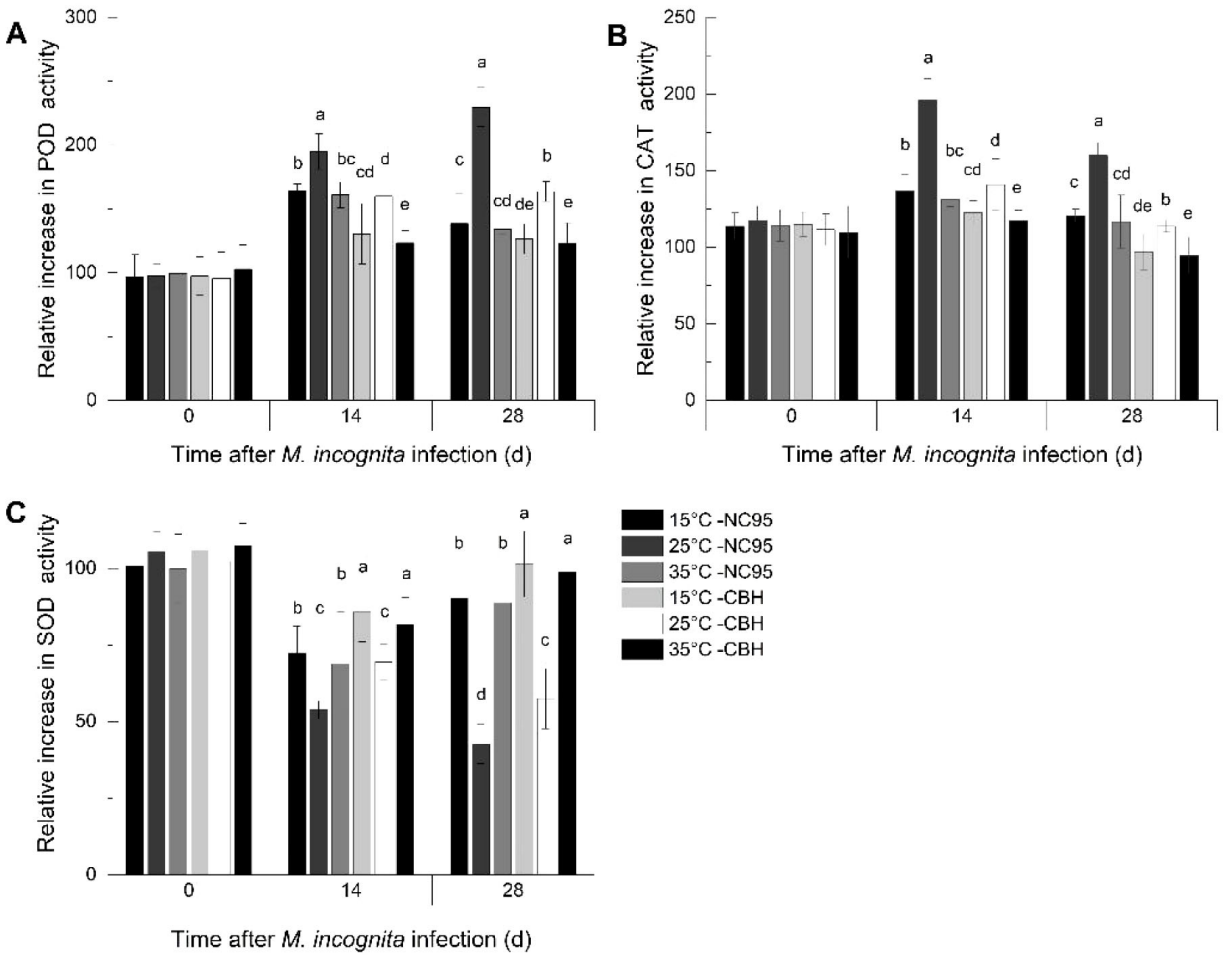
Effects of *Meloidogyne incognita* infection on antioxidant enzyme activities in the roots of two resistant and susceptible tobacco varieties at different temperatures. **(A)** Peroxidase (POD); **(B)** Catalase (CAT); and **(C)** Superoxide dismutase (SOD). Bars with different letters indicate significant differences at *p ≤*0.05.

The relative increase in POD activity in NC95 roots gradually increased at 25°C under *M*. *incognita* infection and showed similar trends at 15°C and 35°C, with peaks appearing at 14 d (**Figure 1A**). The relative increase in NC95 was highest at 25°C and lowest at 35°C throughout the infection period. Compared with NC95 at the same temperatures, the relative increase in POD activity in CBH roots showed a similar trend but remained significantly lower (*p ≤*0.05).

### 3.2 The effects of *M*. *incognita* infection on CAT activity at different temperatures

As shown in **Figure 1B**, the relative increase in CAT activity in the roots of both varieties peaked at 14 d and declined by 28 d across all temperatures, respectively, with the largest change observed at 25°C. In contrast, no significant difference was observed at 15°C and 35°C. Furthermore, the relative increase in CAT activity in NC95 roots was significantly higher than that in CBH at the same temperature throughout the *M*. *incognita* infection period (*p ≤*0.05).

### 3.3 Effects of *M*. *incognita* infection on SOD activity at different temperatures

As illustrated in **Figure 1C**, the relative increase in SOD activity in NC95 roots decreased over time at 25°C, while it was the lowest at 14 d and increased again at 28 d at both 15°C and 35°C. During the *M*. *incognita* infection period, the relative increase in NC95 was lowest at 25°C, with no significant difference observed between 15°C and 35°C. The relative increase in SOD activity in CBH followed a similar trend to that in NC95 at the same temperatures but showed a greater variation during the same period (*p ≤*0.05).

### 3.4 Effects of *M*. *incognita* infection on soluble sugar content at different temperatures

**Figure 2:**
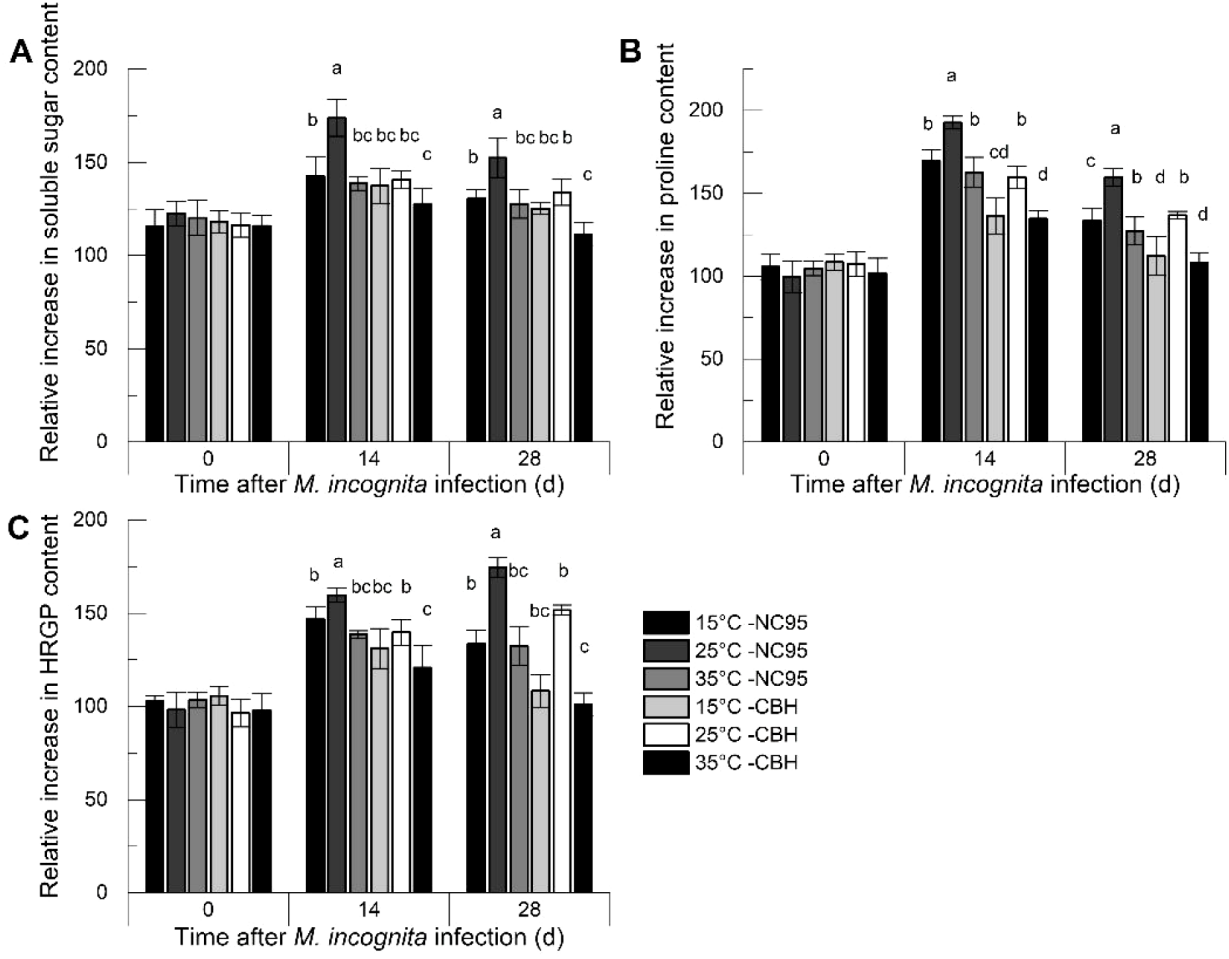
Effects of *Meloidogyne incognita* infection on osmotic regulation substance contents in the roots of two resistant and susceptible tobacco varieties at different temperatures. **(A)** Soluble sugar; **(B)** Proline; **(C)** Hydroxyproline-rich glycoprotein (HRGP). Bars with different letters indicate significant differences at *p ≤*0.05.

As shown in **Figure 2A**, the relative increase in soluble sugar content in both NC95 and CBH roots rose rapidly during the 0–14 d infection period, then declined, with the greatest change observed at 25°C. At 0 d, 14 d, and 28 d, the differences in relative increase between the two varieties at 15°C and 35°C were not significant. Moreover, throughout the entire *M*. *incognita* infection period, the relative increase in soluble sugar content in NC95 roots was significantly higher than that in CBH at the same temperatures (*p ≤*0.05).

### 3.5 Effects of *M*. *incognita* infection on proline content at different temperatures

Under *M*. *incognita* infection (**Figure 2B**), the relative increase in proline content in NC95 roots was highest at 25°C, with no significant difference between 15°C and 35°C. The trend in CBH roots was similar to that in NC95 at the same temperatures; however, the relative increase in proline content was significantly lower during the same period (*p ≤*0.05).

### 3.6 Effects of *M*. *incognita* infection on HRGP content at different temperatures

Under *M*. *incognita* infection (**Figure 2C**), the relative increase in HRGP content in the roots of both varieties was highest at 25°C and lowest at 35°C Additionally, the relative increase in CBH was significantly lower than that in NC95 (*p ≤*0.05). Specifically, the relative increase in content in both NC95 and CBH increased gradually at 25°C, whereas at 15°C and 35°C, peaks occurred at 14 d, followed by a decline.

### 3.7 Effects of *M*. *incognita* infection on root tissue structure at different temperatures

**Figure 3:**
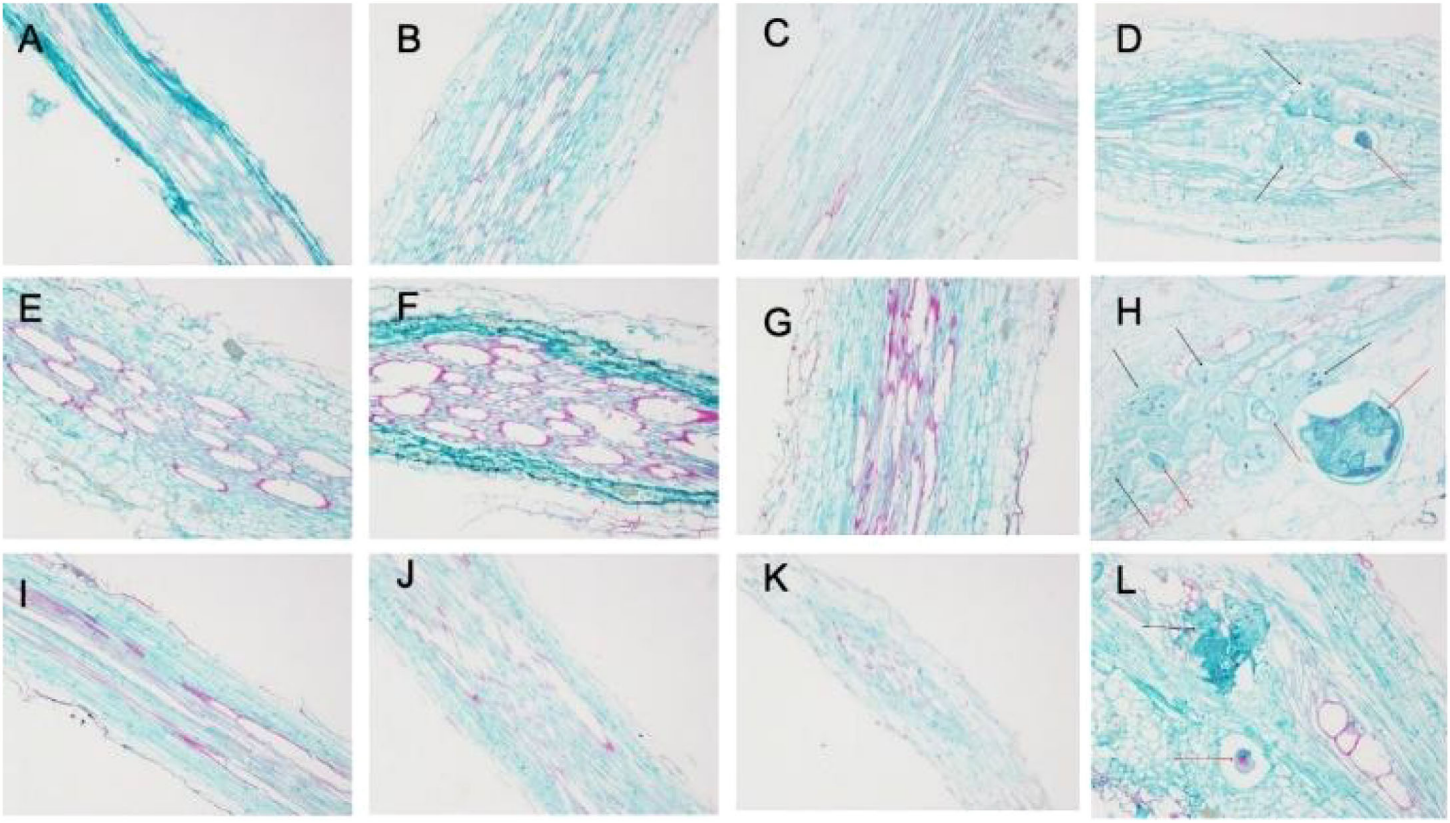
Effects of *Meloidogyne incognita* infection on the root tissue structure of two resistant and susceptible tobacco varieties at different temperatures. **(A)** 15°C–NC95–Control. **(B)** 15°C–NC95–T. **(C)** 15°C–CBH–Control. **(D)** 15°C–CBH–T. **(E)** 25°C–NC95–Control. **(F)** 25°C–NC95–T. **(G)** 25°C–CBH–Control. **(H)** 25°C–CBH–T. **(I)** 35°C–NC95–Control. **(J)** 35°C–NC95–T. **(K)** 35°C–CBH–Control. **(L)** 35°C–CBH–T.

As shown in **Figures 3A, C, E, G, I, K**, there were no differences in the root tissue structure of NC95 and CBH uninoculated with *M*. *incognita* at different temperatures at 28 d post-inoculation. Additionally, vascular bundle tissues showed normal growth and development, with closely arranged xylem and phloem parenchyma and intact cell morphology.

Under *M*. *incognita* infection, the root tissue structure of NC95 showed minimal changes compared with the CK at the same temperature. However, the degree of lignification increased, with the most pronounced lignification observed at 25°C compared to 15°C and 35°C (**Figures 3B, F, J**).

The tissue structure of CBH was seriously disrupted throughout the *M*. *incognita* infection period, exhibiting irregularly arranged xylem and phloem parenchyma and incomplete cell morphology. Meanwhile, oocysts appeared in the xylem (as indicated by the red arrows in **Figures 3D, H, L**), surrounded by giant cells with multiple nuclei (black arrows in **Figures 3D, H, L**), thickened cell walls, darker cytoplasm, and highly irregular morphology and chaotic arrangement. These changes led to severe deformation of xylem structure and ultimately to the formation of root knots, as the local root diameter became abnormally large. In addition, the number of giant cells and oocysts in CBH root tissues was greater at 25°C than at 15°C or 35°C. Fewer giant cells and oocysts were observed at 15°C and 35°C, with no significant difference in their numbers.

### 3.8 Effects of *M*. *incognita* infection on *Rk* gene expression at different temperatures

**Figure 4:**
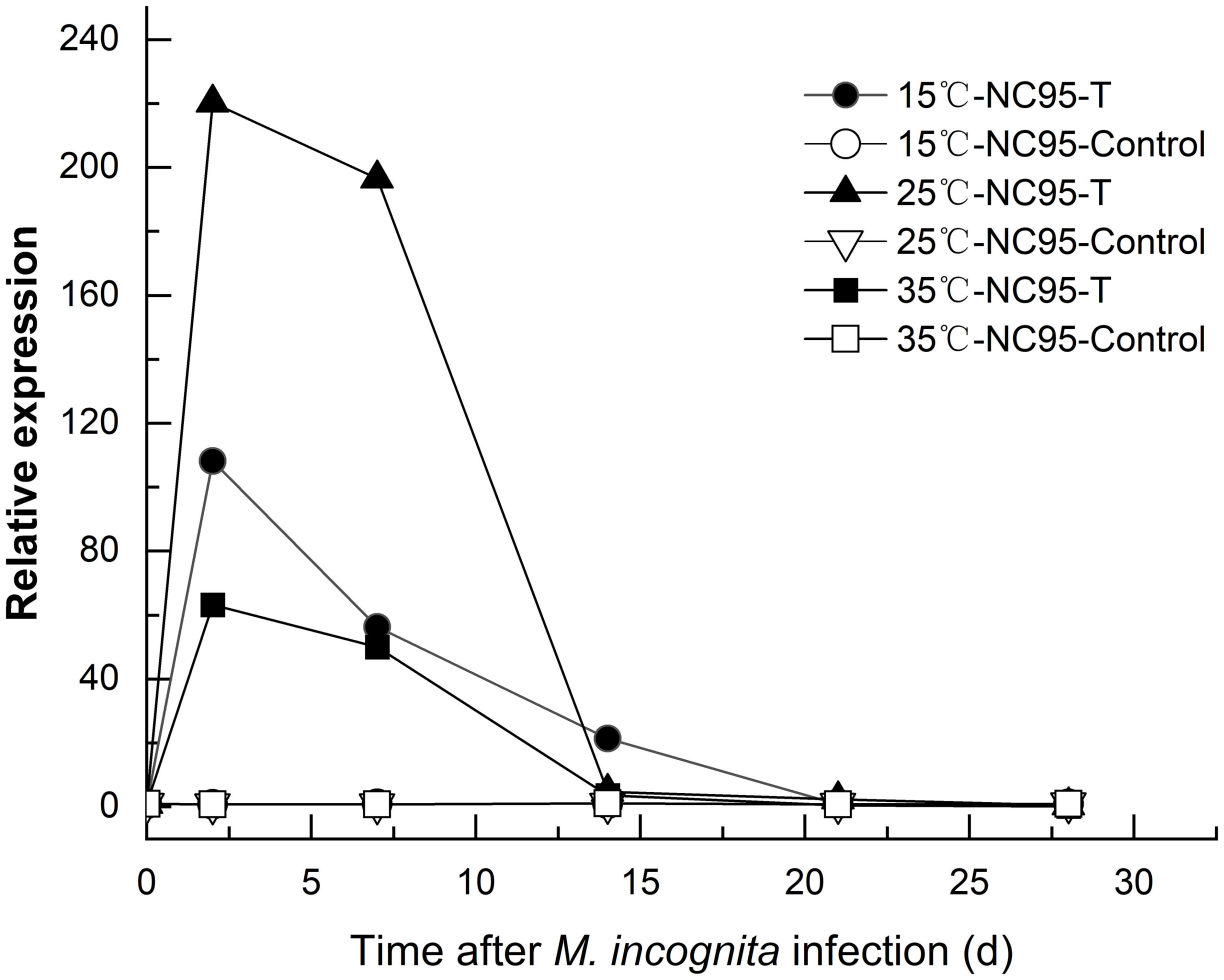
Effects of *Meloidogyne incognita* infection on *Rk gene expression* in the roots of the resistant tobacco variety NC95 at different temperatures.

The results in **Figure 4** indicate that at 15°C under *M*. *incognita* infection, the expression level of the *Rk* gene in NC95 roots first increased and then decreased, reaching a peak at 2 d, which was 108.28 times higher than at 0 d. Furthermore, expression gradually declined from 21 d to 28 d and returned to the initial level by the end of the experiment.

The expression of the *Rk* gene in NC95 was upregulated under infection at 25°C, reaching its highest level at 2 d—220.31 times that at 0 d—before declining under *M*. *incognita* infection.

The expression trend of the *Rk* gene in NC95 at 35°C was similar to that at both 15°C and 25°C under *M*. *incognita* infection. The expression level peaked at 2 d—63.30 times that at 0 d—and then continued to decline. However, under normal growth conditions, the expression level in NC95 remained relatively stable around “1” across all temperatures.

**Figure 5:**
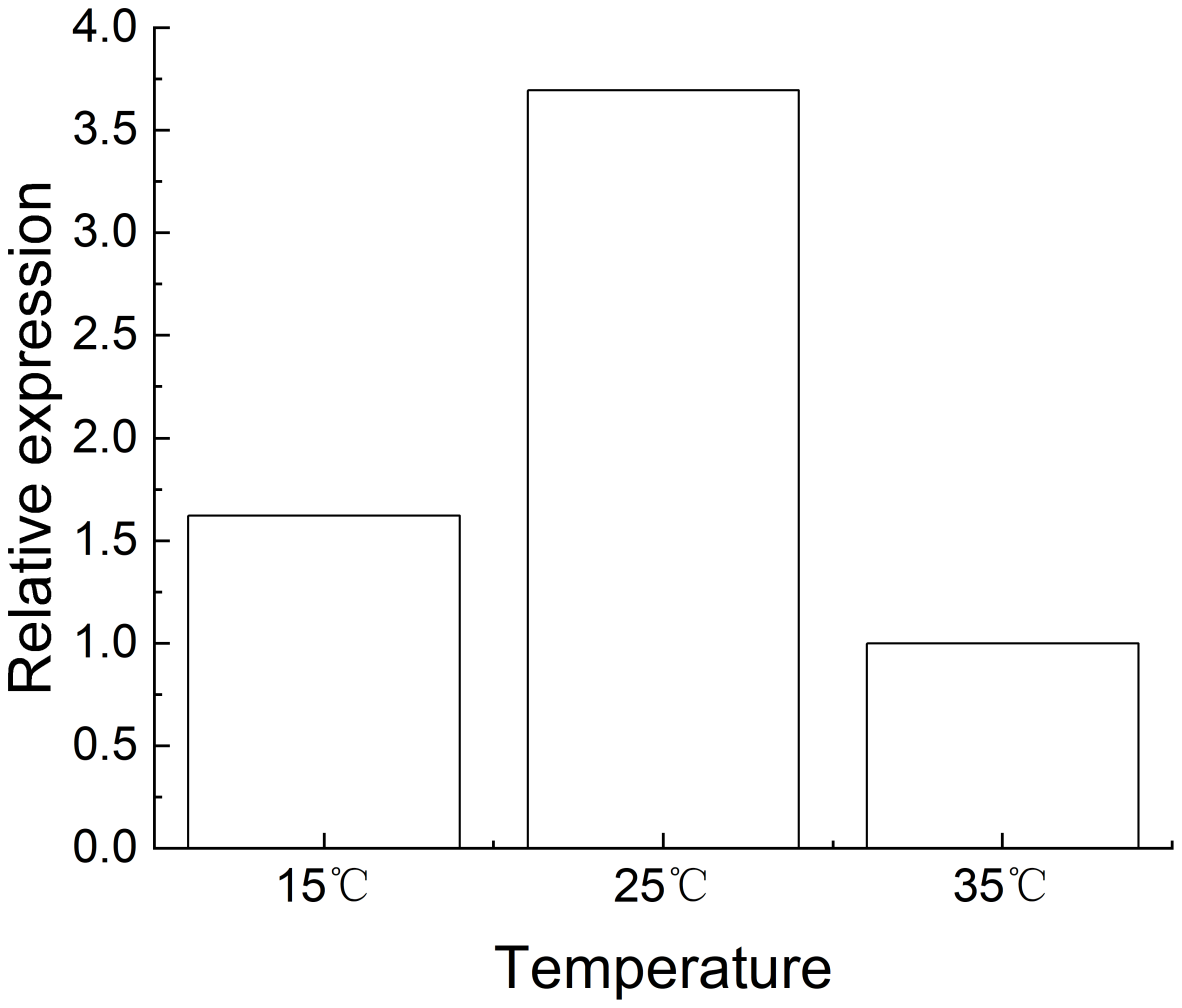
Effects of *Meloidogyne incognita* infection for 2 d on *Rk gene expression* in the roots of the resistant tobacco variety NC95 at different temperatures.

These results indicate that *M*. *incognita* infection led to the upregulation of *Rk* gene expression in NC95 roots, with the greatest increase observed at 2 d. Meanwhile, gene expression was highest at 25°C and lowest at 35°C (**Figure 5**), indicating that 35 °C exerts an inhibitory effect on *Rk* gene expression.

## 4 Discussion

### 4.1 High temperatures attenuate *M*. *incognita* infection by increasing antioxidant enzyme activities and osmotic regulation substance contents

Pathogen infection can increase the levels of reactive oxygen species (ROS) in host cells, making them more vulnerable to oxidative damage. However, the antioxidant enzyme system—of which SOD, POD, and CAT are key components—plays an essential role in scavenging ROS toxicity (Choudhury et al., 2013; Wei et al., 2022a). Normally, these three enzymes function in equilibrium, maintaining ROS production and scavenging at levels that do not harm the plants (Jiang and Huang, 2001; Sugimoto et al., 2014; Zhang et al., 2017). Under pathogen infection, host equilibrium is disrupted, reducing ROS scavenging capacity and resulting in the excessive accumulation of O_2_^•−^ and H_2_O_2_. SOD catalyzes the reaction of O_2_^•−^ with H^+^ to generate O_2_ and H_2_O_2_ (Bafana et al., 2011; Nathan and Ding, 2010) The resulting H_2_O_2_ is then catalyzed by POD and CAT to form O_2_ and H_2_O (Gill and Tuteja, 2010; Khan et al., 2023), thereby reducing the reaction of H_2_O_2_ a O_2_^•−^, a highly reactive species that can damage all cell membranes (Schmitt et al., 2014; Sewelam et al., 2016). In *Dimocarpus longan* infected by *Lasiodiplodia theobromae* (Pat.) Griff. & Maubl. (*L. theobromae*), SOD and CAT activities initially increased and then decreased, leading to ROS accumulation and the loss of resistance (Sun et al., 2018a). Furthermore, in resistant wheat cultivars, SOD and CAT activities increased under *Pyricularia oryzae* infection, and symptoms remained mild (Debona et al., 2012). In addition, tobacco strains overexpressing the *ScCAT2* gene, which encodes CAT from sugarcane, exhibited enhanced resistance to *Ralstonia solanacearum* and *Fusarium solani* var. *coeruleum* (Sun et al., 2018b). Therefore, the activities of SOD, POD, and CAT is closely related to plants stress resistance (Dumanović et al., 2011; Najafi et al., 2024).

Tomato responded to stress through the production of ROS 12 h after *M*. *incognita* infection (Melillo et al., 2006). Zacheo and Bleve-Zacheo (1988) also found that SOD activity was negatively correlated with resistance to *Meloidogyne* spp. in tomato. Furthermore, resistant varieties showed reduced SOD activity under *Meloidogyne* spp. infection, whereas susceptible varieties exhiited higher activity with an upward trend. However, POD activity increased in both resistant and susceptible varieties (Rajasekhar et al., 1997). In contrast, Montes et al. (2004) reported that POD activity significantly increased in resistant wheat varieties under cereal cyst nematode (CCN) infection, accompanied by lower SOD and POD activities in susceptible varieties. In the present study, SOD activity decreased in both tobacco varieties with differing resistance under *M*. *incognita* infection at all tested temperatures. By contrast, POD and CAT activities showed an upward trend, with greater increases in the resistant variety than in the susceptible one, consistent with the findings of Ye et al. (2009). This may be because the increase in O_2_^•−^, influenced by lower SOD activity under *M*. *incognita* infection, triggered hypersensitive responses and ultimately produced toxic effects on *M*. *incognita* (Kerchev and Breusegem, 2022). Meanwhile, higher POD and CAT activities catalyzed H_2_O_2_ into O_2_ and H_2_O, which helped reduce ROS accumulation. Furthermore, increased POD activity contributed to cell wall thickening and lignification, thereby enhancing resistance to *M*. *incognita* infection in roots. Compared to the susceptible variety, POD and CAT activities were higher in the roots of the resistant variety under *M*. *incognita* infection, while SOD activity was lower, indicating stronger ROS scavenging capacity. This contributed to the preservation of root cell structure and function and help maintain physiological balance, ultimately alleviating the damage caused by *M*. *incognita*.

Osmoregulation is an important physiological mechanism by which plants adapt to adverse stress, reducing cellular osmotic potential through the accumulation of osmotic regulatory substances to alleviate dehydration damage to enzymes, organelles, and cell membranes, thereby improving plant resistance (Munns et al., 2022). Soluble sugars and proline are important osmotic regulatory substances in plants (Bai et al., 2019; Su et al., 2021). Furthermore, soluble sugars serve as carbon skeletons and energy sources for the synthesis of organic solutes and play a protective role under high concentrations of inorganic ions in cells (Dai, 2020). In addition, proline is an important component of proteins and often exists in a free state. Under external environmental stress, protein synthesis is inhibited while protein decomposition is promoted, leading to an increase in free proline content to regulate osmotic balance between the cytoplasm and vacuoles (Wei et al., 2022b). Jia (2012) found that proline and soluble sugar contents increased in the roots of different tomato rootstock varieties under *M*. *incognita* infection, with a higher growth rate observed in the resistant variety. HRGP is a major structural component of the cell wall and is closely associated with lignin formation. When infected by pathogens, plants often accumulate large amounts of HRGP to repair the cell wall structure and prevent pathogen penetration, thereby enhancing resistance (Xu et al., 2011; Zeng et al., 2003). Lu (2009) reported that HRGP content in resistant banana varieties was higher than in susceptible ones after *M*. *incognita* inoculation. The present study showed that the contents of soluble sugars, proline, and HRGP in NC95 roots were all significantly higher than in CBH under *M*. *incognita* infection, indicating that root damage induced by *M*. *incognita* could be mitigated by increasing osmotic regulatory substances and strengthening cell wall structure in resistant tobacco varieties.

Temperature is an important climatic factor that affects the growth and development, survival, reproduction, and other life activities of pathogens (Velloso et al., 2022). In addition, appropriate temperature support the normal growth of pathogens, while excessively high or low temperatures are unfavorable for their growth and reproduction (Garbelotto et al., 2021). In our present study, comparison of antioxidant enzyme activities and osmotic regulatory substance contents in two tobacco varieties with different resistance levels under *M*. *incognita* infection at varying temperatures showed that the relative increase of each physiological indicator was greatest at 25°C, while the smallest change occurred at 35°C, indicating that these physiological responses in tobacco roots were less affected by *M*. *incognita* infection at 35°C compared to 25°C. This may be attributed to the increased antioxidant enzyme activities and osmotic regulatory substance contents at higher temperatures, which effectively suppressed *M*. *incognita* infection and reduced its pathogenicity. Conversely, the temperature of 25°C was more favorable for *M*. *incognita* infection in tobacco plants. *M*. *incognita* primarily inhabit the 5 cm–20 cm soil layer. The suitable temperature range for egg hatching and J2 infection is 15°C–30°C, with 27°C being optimal for hatching (Liu, 2000; Wang et al., 2016). Moreover, Chen et al. (2009) reported that temperatures above 35°C inhibited the growth and development of *M*. *incognita* (Chen et al., 2009). Within less than 1 h of treatment at 44°C–45°C, all J2 died, indicating that such high-temperature conditions are detrimental to *M*. *incognita* survival (Wang and McSorley, 2008). Therefore, using double-layer transparent film combined with the addition of organic matter can help increase soil temperature and inhibit *M*. *incognita* in tobacco production (Hou and Liu, 2007). This study offers a new direction for environmentally friendly and sustainable control strategies against *M*. *incognita*.

### 4.2 High temperatures hinder *M*. *incognita* infection by maintaining root tissue structure

Host tissue structure changes following pathogen infection. To some extent, this is an active adaptive response by plants to maintain normal physiological activities; however, it is also a passive reaction that inhibits plant growth and development (Kong et al., 2020). *Meloidogyne* spp. must first penetrate the outer protective layer of roots to infect plants and ultimately cause disease. However, most of J2 are unable to enter the pericycle and remain confined to the root cortical tissue in resistant peanut varieties under *Meloidogyne hapla* infection (Lu et al., 2000). Bendezu and Starr (2003) found that *Meloidogyne arenaria* invaded only the epidermal tissue in resistant varieties, while the susceptible varieties were completely invaded. In contrast, Vovlas et al. (2005) reported that giant cells formed in the roots of *Cordyceps sinensis* across varieties with different resistance levels under *Meloidogyne* spp. infection. Moreover, there was no difference in the number of giant cells. Therefore, it is suggested that the resistance mechanism in resistant varieties involves inhibiting the growth, development, and reproduction of *Meloidogyne* spp. in the roots.

Our present study indicated that no hypersensitive necrosis occurred in the root apical area of either the resistant or susceptible tobacco varieties under *M*. *incognita* infection, showing that the hypersensitive response is not a unique feature of resistant varieties (Lukan et al., 2023). Additionally, there was no significant change in the root tissue structure of the resistant tobacco variety NC95, and no giant cells or oocysts were observed under infection. In contrast, a large number of giant cells and oocysts were observed around the root xylem of the susceptible variety CBH, clearly indicating that different defense mechanisms were present in the resistant and susceptible tobacco varieties under *M*. *incognita* infection. The resistant varieties exhibited a certain degree of immunity to *M*. *incognita* infection, manifested as structural resistance that inhibited the formation of giant cells. This may be because substances secreted by the resistant varieties are toxic to *M*. *incognita* or repel the pathogen from the roots, thereby protecting the plants from infection. Alternatively, the emergence of giant cells and formation of feeding sites may be inhibited by resistant varieties, leading to the death of *M*. *incognita* invading the roots due to nutrients deficiency (Phan et al., 2018), which is consistent with the study by Fan (2020). However, Wang et al. (2006) found that the resistance mechanism in the resistant varieties involved inducing cavitation in giant cells under *M*. *incognita* infection. Additionally, paraffin section analysis of root knots formed by inoculation with *M*. *hapla*, *M*. *javanica* Treub, and *M*. *arenaria* Neal in the resistant wild cucumber variety ‘Hardwickii’ and the susceptible cultivar ‘Smuter’ revealed that elongation of giant cells in resistant varieties resulted in abnormal development of *Meloidogyne* spp (Walters et al., 2006). Collectively, further in-depth research is needed on the root tissue resistance mechanism in the resistant varieties to *M*. *incognita* infection. Moreover, more giant cells and oocysts were observed in the root tissue of the susceptible variety CBH at 25°C than at 35°C. Meanwhile, the degree of lignification in the resistant variety NC95 was also greater at 25°C, indicating that roots maintain better tissue structure at higher temperatures, which may hinder *M*. *incognita* infection.

### 4.3 High temperatures inhibit *M*. *incognita* infection by promoting upregulated expression of *Rk* gene

A complex gene regulatory network is involved in plant responses to adverse stress (Awlia et al., 2021). Under pathogen infection, host resistant responses are induced by the upregulated expression of resistance genes, thereby disrupting the living environment of pathogens and ultimately preventing their growth and development. It has been confirmed that the upregulated expression of resistance genes plays an essential role in the *Meloidogyne* spp.–plant interaction (Gheysen and Fenoll, 2002; Lambert et al., 1999; Vercauteren et al., 2001). To date, several *Meloidogyne* spp. resistance genes have been cloned from crops such as wheat, potato, and tomato. Lagudah et al. (1997) identified the wheat resistance gene of *Cre3* to cereal cyst nematode (CNN). The *Gpa2* gene confers resistance to potato cyst nematode (PCN) infection (van der Vossen et al., 2000). Numerous resistance genes to *Meloidogyne* spp. have been identified in tomato, among which the Mi gene family is the most important, capable of inhibiting nematode development and reproduction (Ernst et al., 2002; Jablonska et al., 2007; Padilla-Hurtado et al., 2022). In addition, the resistance gene *Hs1Pro-1* was discovered in sugar beet by Cai et al. (1997). The expression levels of *MAPK20, ICS1, NPR1*, and *PAD4* were upregulated in the rice resistant variety ‘Phule Radha’ upon infection with *Meloidogyne* spp., whereas no significant expression was observed in the susceptible variety (Hatzade et al., 2020). The combination of *Hs1pro-1* and *cZR3* in rapeseed enhances resistance to *Heterodera schachtii* Schm (Zhong et al., 2019). Furthermore, *Mex-1* from coffee, *CaMi* from pepper, and *RKN1* from cotton had demonstrated effective resistance to *Meloidogyne* spp. infection (Chen et al., 2007; Silva et al., 2013; Wang et al., 2008). In our studies, the resistance gene *Rk* was derived from the *M*. *incognita*-resistant tobacco variety RK42, in which the resistance trait was controlled by a single dominant gene (Pollok et al., 2016; Rufty et al., 1983).

Some resistance genes to *Meloidogyne* spp. exhibit temperature sensitivity, showing complete or partial loss of resistance at elevated temperatures (Hwang et al., 2000; Jablonska et al., 2007). Ammiraju et al. (2003) found that the relative expression of *Mi-1*, *Mi-7*, and *Mi-8* were significantly reduced when resistant tomato varieties were exposed to high temperatures for 1–2 days after inoculation with *Meloidogyne* spp., whereas the expression levels of *Mi-2*, *Mi-3*, *Mi-4*, *Mi-5*, *Mi-6*, and *Mi-9* remain unchanged. The expression profiling of eight *hsp* genes (*Mh-hsp90*, *Mh-hsp1*, *Mh-hsp4*, *Mh-hsp6*, *Mh-hsp60*, *Mh-dnj19*, *Mh-hsp43*, and *Mh-hsp12.2*) in *M*. *hapla* at the egg and J2 stages were highly upregulated under heat stress (at 35°C and 40°C) than under cold stress (at 5°C) (Flis et al., 2024). Consistent with the above studies, our results indicated that the expression of the *Rk* gene in the roots of the resistant tobacco variety roots showed no significant change across temperatures without *M*. *incognita* inoculation; however, its relative expression increased markedly at 2–7 d post infection, indicating that *M*. *incognita* infection promoted the upregulation of *Rk* expression. Moreover, under infection, the expression levels of the *Rk* gene at 25°C and 15°C were 3.36-fold and 1.62-fold higher, respectively than at 35°C. These findings suggest that *Rk* gene expression is temperature-dependent (Pollok et al., 2023), Although expression levels decreased at 35°C, the *Rk* gene was not inactivated and still retained the ability to inhibit *M*. *incognita* infection. Studies on the *Rk* gene remain in its early stages, and further studies are needed to explore how to enhance resistance to *M*. *incognita* infection in tobacco and how temperature affects *Rk* expression.

## Data Availability

The original contributions presented in the study are included in the article, further inquiries can be directed to the corresponding authors.
